# Immunomodulatory effects of the *Bifidobacterium longum* BL-10 on lipopolysaccharide-induced intestinal mucosal immune injury

**DOI:** 10.3389/fimmu.2022.947755

**Published:** 2022-08-24

**Authors:** Jiahuan Dong, Lijun Ping, Ting Cao, Lenan Sun, Deyu Liu, Song Wang, Guicheng Huo, Bailiang Li

**Affiliations:** Food College, Northeast Agricultural University, Harbin, China

**Keywords:** *bifidobacterium longum*, intestinal immunity, immunocyte, NF-κB pathway, Th1/Th2/Th17/Treg, gut microbiota

## Abstract

The intestine is the largest digestive and immune organ in the human body, with an intact intestinal mucosal barrier. *Bifidobacterium longum* is the specific gut commensals colonized in the human gut for boosting intestinal immunity to defend against intestinal mucosal immune injury. In the LPS-induced intestinal injury model, the *Bifidobacterium longum* BL-10 was suggested to boost the intestinal immune. Detailly, compared with the LPS-induced mice, the BL10 group significantly reduced intestine (jejunum, ileum, and colon) tissue injury, pro-inflammatory cytokines (TNF-α, IFN-γ, IL-2, IL-6, IL-17, IL-22, and IL-12) levels and myeloperoxidase activities. Moreover, the *B. longum* BL-10 significantly increased the number of immunocytes (CD4+ T cells, IgA plasma cells) and the expression of tight junction protein (Claudin1 and Occludin). *B. longum* BL-10 regulated the body’s immune function by regulating the Th1/Th2 and Th17/Treg balance, which showed a greater impact on the Th1/Th2 balance. Moreover, the results also showed that *B. longum* BL-10 significantly down-regulated the intestinal protein expression of TLR4, *p*-IκB, and NF-κB p65. The *B. longum* BL-10 increased the relative abundance of the genera, including *Lachnospiraceae_*NK4A136_group and *Clostridia_*UCG-014, which were related to declining the levels of intestinal injury. Overall, these results indicated that the *B. longum* BL-10 had great functionality in reducing LPS-induced intestinal mucosal immune injury.

## Introduction

*Bifidobacterium* is the specific gut commensals colonized in the human gut, especially in the colon. Numerous studies have demonstrated that *Bifidobacterium* is crucial for maintaining the intestinal micro-ecological balance necessary for anti-tumor, immunological modulation, and the deconstruction and transformation of nutrition (1). It also interacts with human immunocytes and modulates specific pathways, involving innate and adaptive immune processes (2). Recent evidence proposed that newborns were born with a sterile gut and their physical immunity increased with age, positively correlated with the number of *bifidobacteria* (3). Thus, *Bifidobacterium* is critical in the maturation of the human immune system from gestation to childhood. This became crucial to understanding the processes by which certain strains might modify the gut microbiota and control immune responses directly or indirectly.

The intestine, directly exposed to disease-causing germs, viruses, and allergens (4), is a critical immunological organ of the body, and its structural and functional characteristics contribute to the formation of the most extensive mucosal immune system (5). Gut-associated lymphoid tissue (GALT) is an essential part of the intestinal mucosal immune system (6), and it is also the leading site of mucosal immunity, which consists mainly of mesenteric lymph nodes (MIN), lamina propria (LP), Peyer’s patch (PP), and intestinal epithelial lymphocytes (IEL) (7). In detail, the initial line of intestinal defense is based on intestinal epithelial cells secreting antimicrobial peptides (AMP) termed defensins (8). The second line of defense is the creation of immunoglobulin A (IgA) that may pass the epithelial barrier between the host and the microbiota (9), completely covering each bacterium and blocking bacterial translocation. The secretory immunoglobulin A (sIgA) is an essential antibody on the mucosal surface exerting immune clearance without inflammatory immune response (10). The sIgA prevents intestinal infection by conditionally harmful bacteria by binding to antigens and blocking adhesion and invasion (11). SJOGREN et al. demonstrated that *Bifidobacterium bifidum* increased sIgA secretion in intestinal mucosa by measuring sIgA levels in stool and saliva specimens from infants (12). The third line of defense consists mainly of immune cells associated with it, such as dendritic cells (DC), B lymphocytes, T lymphocytes, and innate lymphocytes (13). Previous studies revealed that *Bifidobacterium* might directly modulate the immune system by interacting with GALT.

*Bifidobacterium* has a protective effect on the intestinal mucosa in two ways (14), involving maintaining the balance of the intestinal microflora (14) and directly acting on the immune system to induce intestinal immunity. Yan et al. confirmed that *Bifidobacterium bifidum* YS108R fermented milk partially reversed the imbalance of gut microbiota in DSS-induced mice and prevented the increase of pathogenic bacteria (15). Further, *Bifidobacterium* can stimulate intestinal mucosal immune cells to secrete cytokines, with an important role in inducing proliferation and differentiation of immune cells and enhancing the immune response. The growth of T cells in the thymus was affected by *Bifidobacterium via* enhancing the maturation of regional dendritic cells and IL-12 expression in the gut, according to the research (16).

*Bifidobacterium* can suppress the expression of toll-like receptor 4 (TLR4), regulate T cell differentiation, and inhibit the NF-κB pathway activity by improving the intestinal immune response to pathogenic bacteria (17). Previous researches on the relationship between it and immune cells pointed to *Bifidobacterium* treatment mice stimulating DCs proliferation and immunological activation by increasing DCs numbers and anti-tumor CD8+ T cell proliferation (18). Moreover, *Bifidobacterium* can improve the mucosal immune system by indirectly controlling the gut microenvironment (19). For instance, DONG et al. demonstrated that *Bifidobacterium* could enhance intestinal immune function, as it promoted IL-12 secretion from intestinal dendritic cells, up-regulated IL-10 and IFN-γ secretion in plasma, increased the IFN-γ/IL-4 ratio in the intestinal mucosa, and promoted thymic T cells differentiation toT helper cells 1 (Th1) (16).

*Bifidobacterium longum* BL-10, a newly identified probiotic, was obtained from the feces of healthy breast-fed infants in the key lab of dairy science (KLDS). In a recent study, *B. longum* BL-10 possessing beneficial intestinal colonization properties was the most effective promoter of proliferation on normal colon epithelial CCD 841 CoN cells (20). *In vitro* experiment, we evaluated its effect on an LPS-induced cell injury model and showed that *B. longum* BL-10 could promote intestinal development by facilitating human fetal colon epithelial cell proliferation and accelerating the maturation of the internal barrier (20). However, the immunoregulatory properties of *B. longum* BL-10 remain unknown *in vivo*, as is its immunoregulatory mechanism. In this study, we selected the LPS-induced BALB/c mice model to explore the mechanism of *B. longum* BL-10 to regulate intestinal immunity. We also investigated the cytokines and immune cell regulatory responses of *B. longum* BL-10 treatment on pro-inflammatory cytokine levels (TNF-α, IL-4, IL-6, and IL-12) and key regulatory protein expressions. This study provides a theoretical basis for *B. longum* practical applications, aiming to develop probiotic preparations for enhancing immunity.

## Materials and methods

### Bacterial isolates and culture conditions

*B. longrum* BL-10 was a novel strain of bacteria from the intestines of healthy infants and *Bifidobacterium animalis subsp. lactis* BB-12 was purchased from Chr. Hansen Inc (Hoersholm, Denmark). All the experimental strains were stored at the Key Laboratory of Dairy Science (KLDS) of Northeast Agricultural University (NEAU, Harbin, China). For three generations, they were incubated in the MRS (de MAN, ROGOSA, and SHARPE) Broth with 0.05% L-cysteine (AOBOX, Beijing, China) at 37°C for 24 h in an anaerobic condition. After cryogenic centrifugation at 8000 rpm for 10 min, cell pellets were extracted and washed twice with physiological saline (0.9% NaCl). As to being administered intragastrically, the experimental strains were adjusted to 1.0×10^9^ CFU/mL and stored at 4°C.

### Animals and experimental design

**Figure 1:**
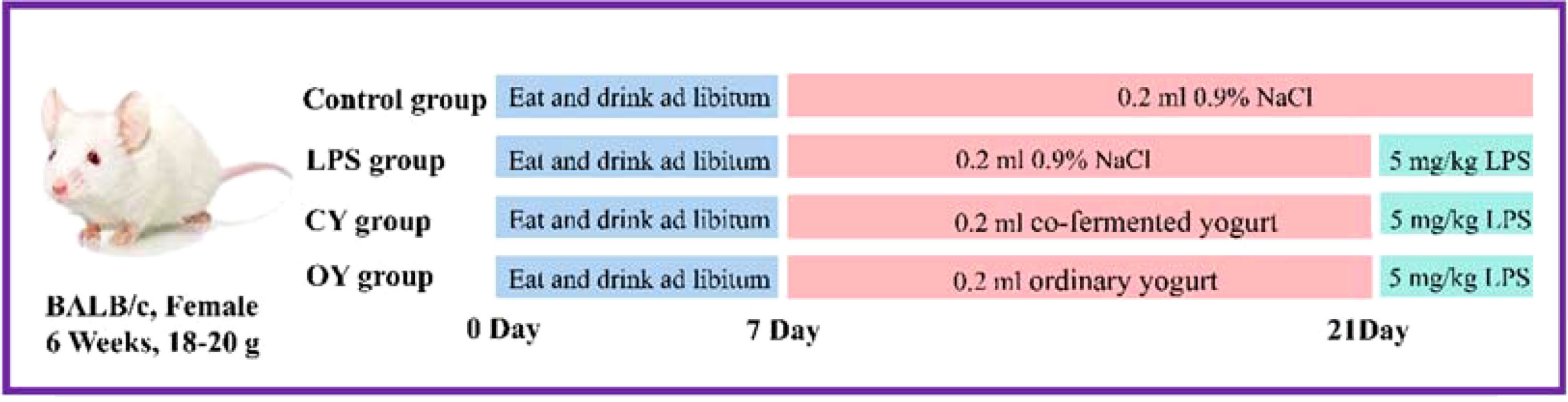
Animal model experimental design.

40 BALB/c mice (SPF, female, six weeks old, 18–20 g) were obtained from the Beijing Vital River Laboratory Animal Technology Co. Ltd (Beijing, China). For experimental mice, they were acclimated to the laboratory environment at Northeast Agricultural University (Harbin, China) for at least one week. The environment maintained pathogen-free at constant temperature (20°C ± 2°C) and humidity (50% ± 5%) with 12 h/12 h light/dark cycle, access to water, and standard food. The experiment was authorized by the Animal Ethics Committee of NEAU (ethic approval code: NEAUEC20210475). **Figure 1** depicts the detailed animal experiment. Briefly, all the mice were randomly divided into four groups (n=10), including the Control group, LPS group, BL10 group, and BB12 group. The mice in the control and LPS groups were intragastric with 0.2 mL saline daily, and the other two groups received 0.2mL bacteria suspension of *B. longrum* BL-10 and *Bifidobacterium animalis subsp.lactis* BB-12 (1.0×10^9^ CFU/mL), respectively. After 14 days, laboratory mice except the control group were injected intraperitoneally at 5 mg/kg body weight and carried out euthanasia after 6 hours.

### Hematoxylin-eosin stain

The intestinal tissues were treated for histological observation as the method described previously (21). Briefly, the jejunum, ileum, and colon samples were collected, fixed by immersion in 4% formaldehyde, and embedded in paraffin. Subsequently, the paraffin slices were placed on slides and stained with hematoxylin and eosin (H&E). Histological examination was performed using Microscopy BX53 (OLUMPUS, Japan) at × 200 magnification. Each mouse’s disease activity index (DAI) and histological score (HS) were evaluated based on the standards (**Supplementary Tables 1**, **2**) to assess the degree of intestinal injury.

### Alcian blue-periodic acid sthiff stain

The AB-PAS stain revealed the presence of goblet cells and mucin in the colon and ileum. The staining solution was dripped over the samples’ paraffin sections, and the sections were subsequently washed in water. Afterward, the slices were rinsed with tap water and re-stained with hematoxylin. All samples were observed and photographed using the Microscopy BX53 (OLUMPUS, Japan) at × 200 magnification. The results of Chiu’s score were assessed in light of the factor (**Supplementary Table 3**).

### Intestinal immunohistochemistry

The immunohistochemistry (IHC) method was performed as previously described by Liu (22). The paraffin-embedded ileac sections were sliced to a thickness of 4 μm and placed on a slide. After being deparaffinized in xylene and rehydrated through ethanol to water, the slides were washed in PBS-0.1% (v/v) Tween 20 and dried. Endogenous peroxidase activity was suppressed for 15 min with 3% hydrogen peroxide in distilled water diluted with PBST. Following antigen extraction, the standard goat serum working solution was used to inhibit the non-specific antibody binding sites for 1 hour. The sections were respectively incubated with one of the following primary antibodies: rabbit anti-CD4+ (1:200, Abcam, Cambridge, UK), rabbit anti-CD8 alpha (1:200, Abcam), and rabbit anti-CD209 mAb (1:200, ABclonal, Wuhan, China). The slices were incubated for 30 min at 25°C with goat anti-rabbit IgG (1:400) from Millipore (Billerica, MA, USA) and observed by fluorescence microscopy at × 200 magnification. The outcomes were measured according to the Frenchy’s method (23), detailly, the mean gray value (staining intensity) and positive area percentage (staining area) of positive cells were used as IHC measurement indexes with high positive (3+), positive (2+), low Positive (1+) and negative (0).

### Intestinal Immunol fluorescence

The detection of IgA-producing plasma cells on the ileum was assayed by direct immunofluorescence (IF) with the method of de Moreno et al. (24). The tissue sections were blocked with normal goat serum working solution for 10 minutes and incubated with Goat Anti-Mouse IgA alpha chain (Biotin) (1:200, Abcam) for 1 hour at 25°C. Each sample was treated with donkey anti-goat IgG (H+L) (1:150, Abcam) and photographed under a fluorescence microscope. The result was expressed as the photograph’s mean gray value (integrated density/area).

### Enzyme-linked immunosorbent assay

The 2% tissue samples homogenized in saline (w/v=1:49) were prepared to determine IFN-γ, TNF-α, TGF-β, IL-2, IL-4, IL-6, IL-10, and IL-22 levels. The serum samples were left to stand at room temperature for 30 min and isolated by centrifugation at 4°C (3500 r/min, 10 min) to measure MPO activity and D-lactate (D-Lac) activities. All the samples were detected using commercial ELISA kits (Conodi creatures, Fujian, China) by following the kit instructions.

### Quantitative RT-PCR

The mRNA expression levels of the major tight junction (TJ) protein ZO-1, Claudin1, Occludin, and mucoprotein mucin (Muc) 2 were investigated by RT-qPCR. Total RNA was obtained from ileac tissues by the RNAiso Plus kit, and cDNA was generated by the Transcriptor First Strand cDNA Synthesis kit (Vazyme, Nanjing, China). The PCR reactions were performed by Stormstar SybrGreen qPCR Master Mix (Promega, Madison, USA) on the Go Taq^®^ SYBR-Green qPCR Master Mix (Promega, Madison, USA). Especially, in this study, the β-actin was used as the internal reference gene (**Supplementary Table 2.3**).

### Western blot

The total protein was extracted from the appropriate ileac tissue regarding the kit method and was determined *via* the BCA kit. The Western blot assay was referenced from Xie et al. (25). Briefly, 30 μg issue was placed on a 10% SDS-PAGE gel to separate the proteins in each well. Western blots were blocked in 5% bovine serum albumin (BSA) and produced in Tris-buffered saline containing PBS-0.1% (v/v) Tween 20 for 2 hours at room temperature (TBST). Subsequently, all the samples were treated overnight at 4°C with rabbit polyclonal antibodies: anti-T-bet, anti-GATA-3, and anti-β-actin. After incubation with secondary HRP-conjugated goat antirabbit IgG, the result was analyzed by Gel-Pro-Analyzer software and normalized to the β-actin and Histone H3.

### 16S rRNA gene of gut microbiota

The gut microbiota total DNA was extracted according to the instructions of the fecal DNA extraction kit, a mix of the samples was based on the amount of data produced and the size of the fragments. Then samples were sequenced in the third generation on the PacBio sequencing platform. The effective-CCS sequence was 30 clustered according to the similarity of 97.0% to obtain OTUs. Finally, the diversity, difference, relevance, and species classification of the gut microbiota were analyzed based on the composition of the OTU sequence.

### Statistical analysis

The result was analyzed using SPSS 25.0 software (SPSS Inc., Chicago, IL, USA), and assessed *via* independent sample T-test (Independent t-test) and the One-way ANOVA (Tukey’s tests). The significance level was set at p < 0.05, and the graphs were generated with GraphPad Prism 9.3 software (GraphPad Inc., La Jolla, CA, USA).

## Results

### *B. longum* BL-10 treatment improves the pathological changes in mice

**Figure 2:**
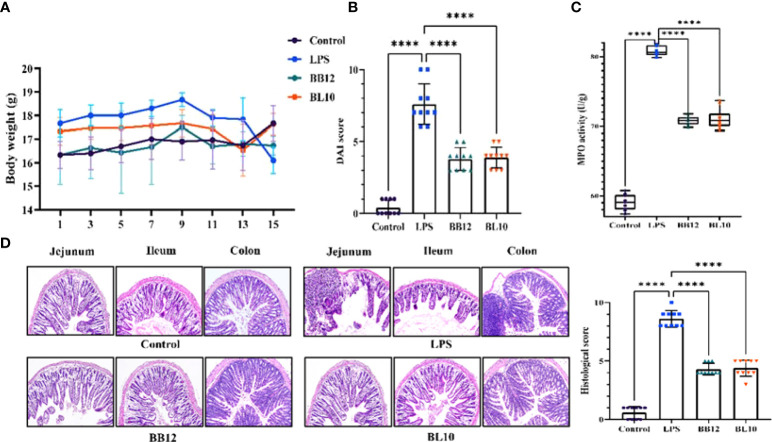
Effect of *B longum* BL-10 on LPS-induced intestinal immune injury. **(A)** Body weight; **(B)** DAI score; **(C)** MPO activity; **(D)** H&E staining (magnification × 200) and colon histological score. All data are expressed as mean ± SD (n=10). Values with a different superscript letter (****) were significantly different at *p*< 0.0001.

*Bifidobacterium* treatment had no palpable toxic side effects on mice as the bodyweight of mice gradually increased and the difference among all groups was significant after intraperitoneal injection. The disease activity index (DAI) score and MPO activity suggested intestinal injury levels were significantly (*p*<0.05) increased in the LPS group, while those were significantly (*p*<0.05) decreased in the BL10 group. **Figure 2D** was the result of H&E staining to reflect the tissue damage in the jejunum, ileum, and colon, which reflected the pathological changes to some extent. In the control group, the intestinal villi were neatly arranged, epithelial cells were intact, and the tissue structure was clear, without edema and inflammation. In contrast, the tissue in the LPS group had obvious edema, intestinal wall congestion, shortened intestinal villi, loss of glands, and infiltration of inflammatory cells. Treatment with *Bifidobacterium* recovered the intestinal injury compared to the LPS group, including improving the morphological structure of the intestine and reducing the inflammation level in mice, with *B. longum* BL-10 being the most significant. Meanwhile, the histological score of mice was investigated, and the significant increase was in accord with the H&E staining (**Figure 2D**).

### *B. longum* BL-10 treatment recovers the barrier function recover in mice

**Figure 3:**
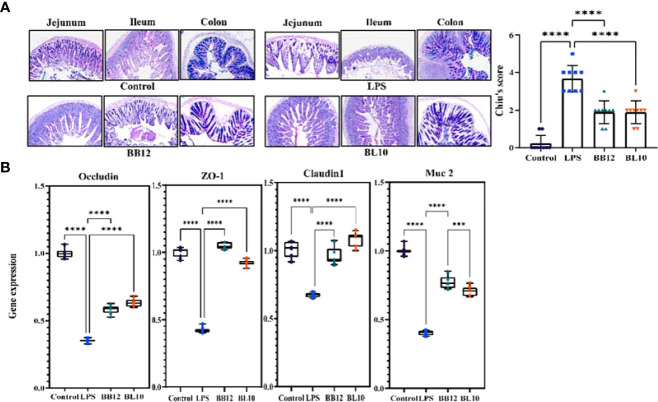
Effect of *B longum* BL-10 on LPS-induced intestinal barrier protection. **(A)** AB-PAS staining (magnification × 200); **(B)** Tight junction protein mRNA expression. All data are expressed as mean ± SD (n=10). Values with a different superscript letter (*** and ****) were significantly different at *p*< 0.001 and *p*< 0.0001.

The AB-PAS staining was done to clearly identify the intestinal goblet cells and mucin production, and the intestinal Chiu’s score showed how badly the gut barrier had been damaged. In **Figure 3A**, there is a clear trend of increase (*p*<0.05) in Chiu’s score and a sharp decrease in mucin secretion of the LPS group compared with the control group. Further indicators of intestinal barrier function, including TJ protein (Claudin1, Occludin, and ZO-1) and mucin 2 (Muc 2), were substantially reduced (*p*<0.05) in the LPS group, hinting barrier function injury (**Figure 3B**). In contrast, the *Bifidobacterium* groups increased the expression of mRNA in each indicator, and *B. longum* BL-10 was preferable to increase expression levels of Claudin1 and Occludin (*p*<0.05). Overall, these results indicated that *B. longum* BL-10 effectively alleviated the reduced intestinal barrier function caused by LPS.

### *B. longum* BL-10 treatment increases the IgA plasma cells score in the ileum

**Figure 4:**
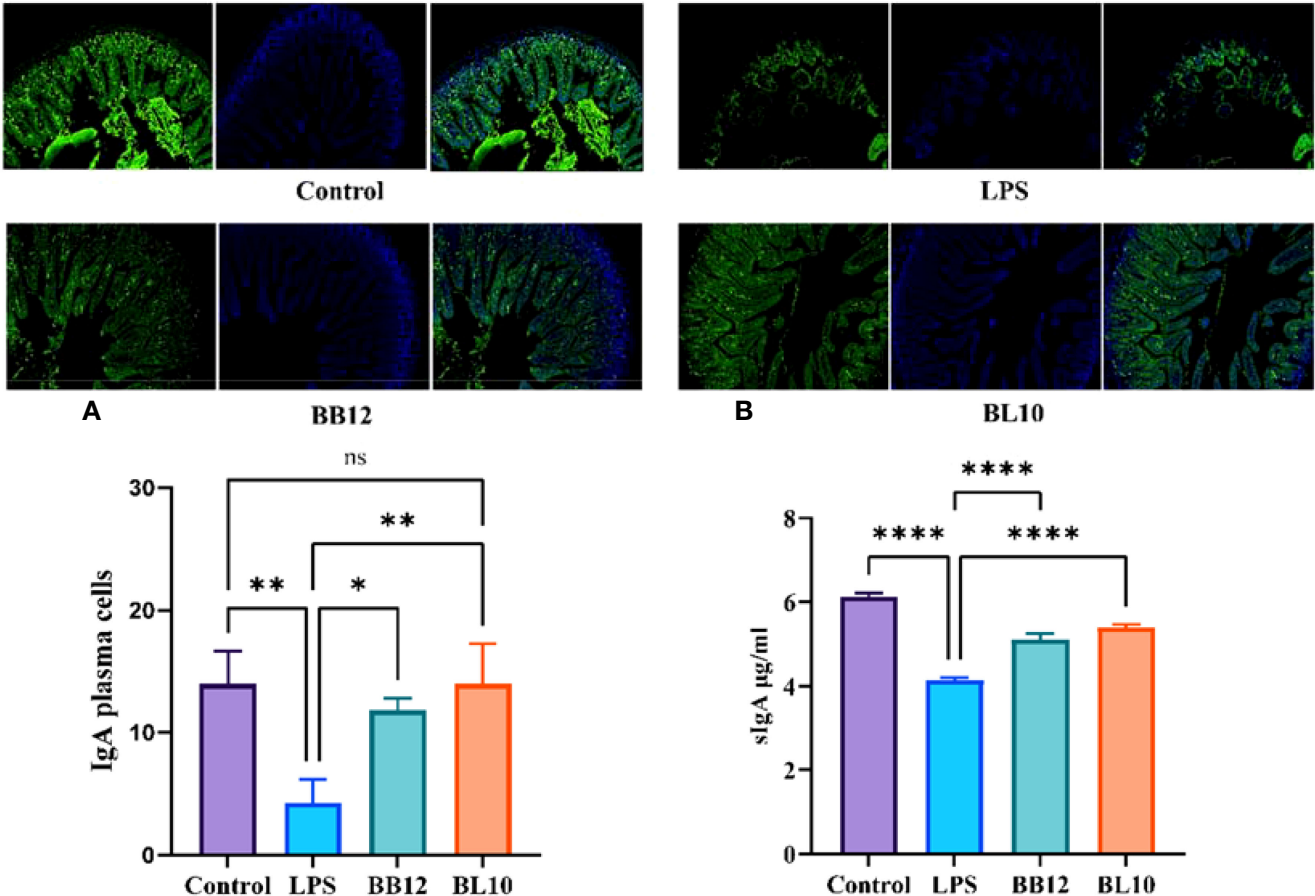
Effect of *B longum* BL-10 on ileal IgA plasma cells. **(A)** IgA plasma cells; **(B)** sIgA level. All data are expressed as mean ± SD (n=3). Values with a different superscript letter (*, **, **** and ns) were significantly different at *p*<0.05, *p*<0.01, *p*<0.0001 and *p*>0.05.

Activated B cells in the lamina propria develop into IgA plasma cells, which release sIgA to boost the antibody response. After intraperitoneal injection of LPS, IgA plasma cells were substantially reduced (*p*<0.05) in the LPS group, indicating that LPS inhibited the cell differentiation of B cells to IgA plasma cells (**Figure 4A**). The scores of IgA plasma cells in the *Bifidobacterium* groups were significantly increased (*p*<0.05) compared with the LPS group, and *B. longum* BL-10 preferably increased it. Moreover, as sIgA was an important secretory immunoglobulin secreted by IgA plasma cells, its level further reflected the intestinal mucosal immunity (**Figure 4B**). Compared with the LPS group, *Bifidobacterium*, including *B. animalis subsp. lactis* BB-12 and *B. longum* BL-10 markedly increased (*p*<0.05) the sIgA level, especially in the BL10 group. Our findings showed that *B. longum* BL-10 might enhance gut immunity by promoting IgA plasma cell proliferation and differentiation.

### *B. longum* BL-10 treatment increases immune cells score in the ileum

**Figure 5:**
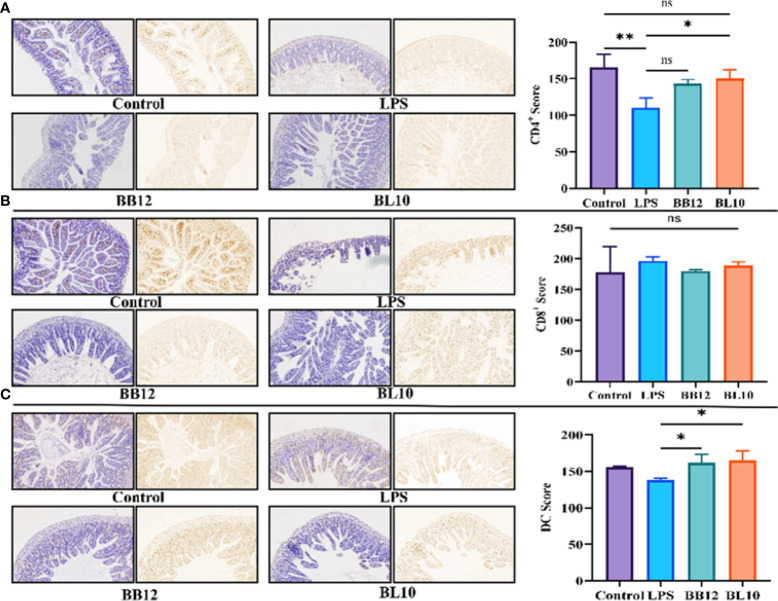
Effect of *B longum* BL-10 on ileal DCs, CD4+ and CD8+ T cells. **(A)** CD4^+^ T cells; **(B)** CD8^+^ T cells; **(C)** DC cells. All data are expressed as mean ± SD (n=3). Values with a different superscript letter (*, ** and ns) were significantly different at *p*<0.05, *p*<0.01 and *p*>0.05.

T cells divided into CD4^+^ and CD8^+^ T cells were the main immunocyte of the immune response and immune regulation. In this experiment, immunosuppression did not influence the CD8^+^ T cell population since *B. longum* BL-10 treatment had comparable CD8^+^ T cell counts to the control and LPS groups (**Figure 5B**), and there were no significant changes (*p*>0.05) in any of the groups. Nevertheless, compared with the Control group, the number of CD4^+^ T cells in the LPS group was significantly decreased (*p*<0.05). After intragastric injection of *Bifidobacterium*, the CD4^+^ T cells count increased significantly (*p*<0.05) in the BB12 and BL10 groups, with the BL10 group having the greatest potential to boost CD4^+^ T cells (**Figure 5A**). Moreover, the intensity of immunity of the intestinal mucosa can also be reflected in the number of DC cells. As shown in **Figure 5C**, compared with the Control group, the number of DC cells in the LPS group was slightly reduced. However, compared to the LPS group, the *Bifidobacterium* treatment groups significantly increased (*p*<0.05) the number of DC cells, with the best results for the BL10 group (*p*<0.05). These results showed that *B. longum* BL-10 improved intestinal immunity by increasing the number of immunocytes, including CD4^+^ T cells and DC cells.

### *B. longum* BL-10 treatment adjusts cytokines secretion in the ileum

**Figure 6:**
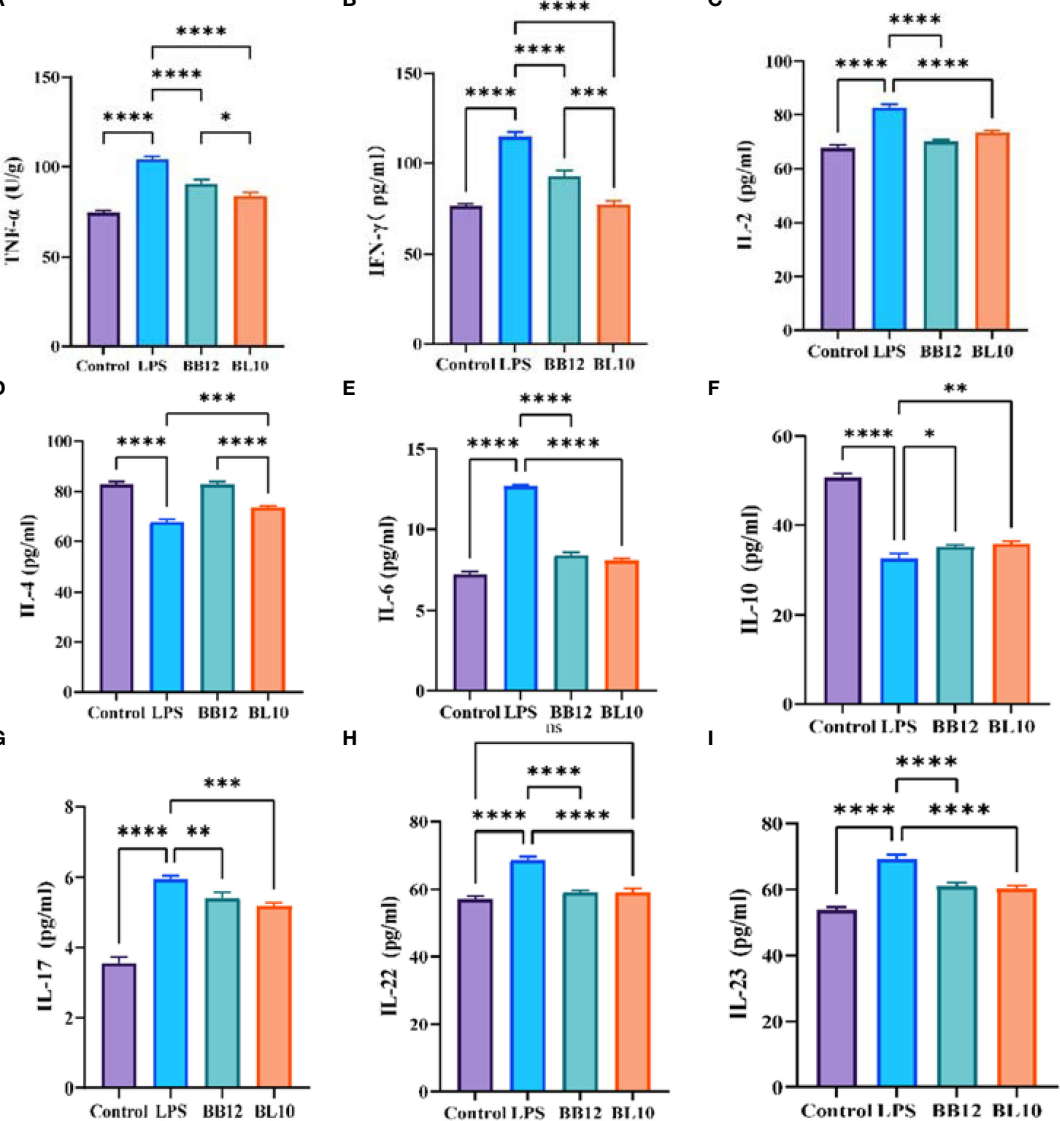
Effect of *B longum* BL-10 on ileal inflammatory factor. **(A)** TNF-α; **(B)** IFN-γ; **(C)** IL-2; **(D)** IL-4; **(E)** IL-6; **(F)** IL-10; **(G)** IL-17; **(H)** IL-22; **(I)** IL-23. All data are expressed as mean ± SD (n=3). Values with a different superscript letter (*, **, ***, **** and ns) were significantly different at *p*<0.05, *p*<0.01, *p*<0.001, *p*<0.0001 and *p*>0.05.

The cytokines, transmitting biological information between cells, were used to indicate intestinal mucosal immunity (**Figure 6**). *B. longum* BL-10 treatment significantly reduced (*p*<0.05) the levels of TNF-α, IFN-γ, and IL-2, which belonged to Th1 type dominant cytokines. Th2 type dominant cytokines, including IL-4, IL-6, and IL-10, were also altered in the experimental groups, where the proinflammatory cytokine IL-6 was significantly increased (*p*<0.05) and anti-inflammatory cytokines (IL-4 and IL-10) were significantly reduced (*p*<0.05) in the LPS group. *B. longum* BL-10 treatment substantially reversed adverse circumstances, especially in increasing IL-4 levels. Moreover, treatment with *B. longum* BL-10 significantly altered the cytokine profile in the ileum, namely the cytokines IL-17, IL-22, and IL-23, which were associated with Th17 and Treg cells’ immunological responses, respectively. After giving a gavage of *B. longum* BL-10, the levels of IL-17, IL-22, and IL-23 were evidently reduced (*p*<0.05) compared to the LPS group. These results suggested that *B. longum* BL-10 affected cytokine secretion on the injury of intestinal mucosal immuno-barrier function.

### *B. longum* BL-10 treatment adjusts the key protein expression levels in the ileum

**Figure 7:**
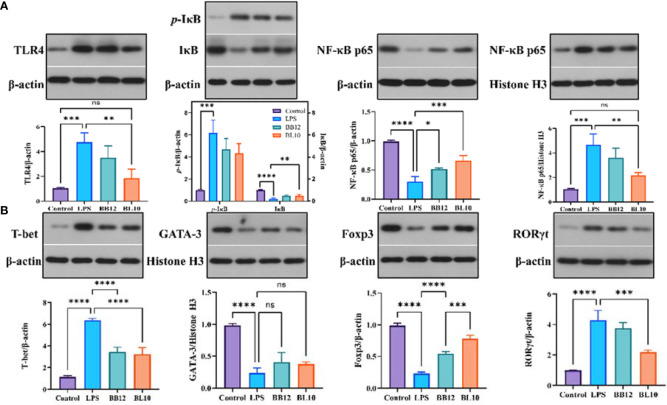
Effect of B. longum BL-10 on gut microbiota composition. **(A)** PCA; **(B)** Gut microbial changes at the phylum level; **(C)** Gut microbial changes at the genus level; **(D)** Prediction of the gut microbiota function; **(E)** Correlation analysis about gut microbial alteration. All data are expressed as mean ± SD (n=3). Values with a different superscript letter (*, **, ***, **** and ns) were significantly different at p<0.05, p<0.01, p<0.001, p<0.0001 and p>0.05.

LPS activated intracellular NF-κB to produce large amounts of pro-inflammatory factors, triggering an inflammatory cascade in intestinal mucosal immunity. The protein expression levels of ileac TLR4, IκB, and NF-κB p65 in the LPS, KLDS BL-10, and BB12 groups were detected and the outcomes were shown in **Figure 7**. LPS resulted in a significant rise (*p*<0.05) of key proteins (TLR4, *p*-IκB, and NF-κB p65) in the NF-κB signaling pathway, while the IκB expression was significantly reduced (*p*<0.05) compared with the control group. Supplementation of *B. longum* BL-10 dramatically decreased (*p*<0.05) the expression of cytoplasmic TLR4, *p*-IκB in and nuclear NF-κB p65, and the cytoplasmic IκB and NF-κB p65 were significantly increased (*p*<0.05). Furthermore, the *B. longum* BL-10 had a more significant effect compared to the BB12 group. Moreover, the protein levels of specific transcription factors T-bet, GATA-3, RORγt, and Foxp3 derived from Th1 cells, Th2 cells, Th17 cells, and Treg cells were examined by Western blotting. The result showed that the levels of GATA-3 and Foxp3 in the Control and BL10 groups were significantly higher (*p*<0.05) than that of the LPS group, while the LPS group was significantly increased (*p*<0.05) the T-bet and RORγt levels compared with the control group. Additionally, the T-bet/GATA-3 ratio in the BL10 group was declined by upregulated transcriptional activity of Th2 cells and downregulated the transcriptional activity of Th1 cells. The outcomes also manifested that *B. longum* BL-10 downregulated the ratio of RORγt/Foxp3 to recover the balance of Th17 and Treg cells. To summarize, *B. longum* BL-10 could prevent the injury of intestinal mucosal immunity *via* regulating the balance of immunocytes and inhibiting inflammation.

### *B. longum* BL-10 treatment recovers gut microbiota imbalance

**Figure 8:**
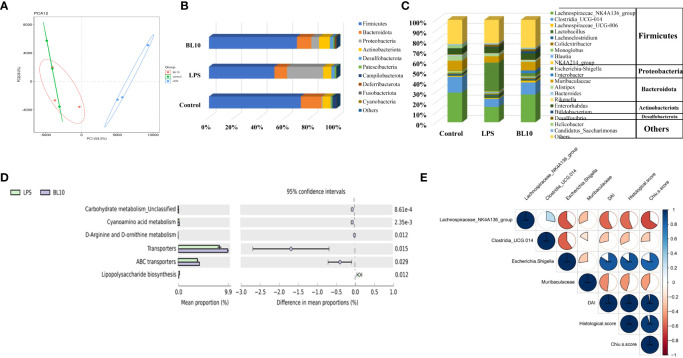
Effect of *B longum* BL-10 on NF-κB signaling pathway and immune key protein. **(A)** NF-κB signaling pathway; **(B)** Immune key protein. All data are expressed as mean ± SD (n=3). Values with a different superscript letter (*, **, ***, **** and ns) were significantly different at *p*<0.05, *p*<0.01, *p*<0.001, *p*<0.0001 and *p*>0.05.

PCA denoted the discrepancy in gut microbial composition between all the groups (**Figure 8A**), and the composition in the BL10 group was similar to the Control group. Three experimental groups were divided into different clusters by PC1 (53.2%) and PC2 (26.2%) influence factors, suggesting the differences in gut microbiota. Firmicutes, Bacteroidota, Proteobacteria, and Actinobacteriota were prime bacteria in the three groups (**Figure 8B**). Firmicutes (51.35%) and Bacteroidota (9.90%) declined but Proteobacteria (28.41%) increased in the LPS group versus the Control group (Firmicutes, Bacteroidota, and Proteobacteria were 72.09%, 16.26%, and 0.82%, respectively). The BL10 group increased Firmicutes (17.70%) and Bacteroidota (1.35%) and reduced Proteobacteria (22.32%), compared to the LPS group. As shown in **Figure 8C**, the histogram explained the relative abundance of microorganisms at the genus level. Compared with the Control group, *Lachnospiraceae_NK4A136_group*, *Clostridia_UCG-014* and *Muribaculaceae* were reduced to 15.06%, 7.81%, and 6.22%, respectively, but *Escherichia-Shigella* was increased to 28.02%. *B. longum* BL-10 treatment recovered gut microbiota imbalance by reversing these changes in the gut. To infer the distinct gene functions between the LPS and BL10 groups, **Figure 8D** performed that carbohydrate metabolism Unclassified, cyanoamino acid metabolism, D-arginine and D-ornithine metabolism, transporters, and ABC transporters were higher in the BL10 group than these in the LPS group, but lipopolysaccharide biosynthesis was lower. Based on the correlation analysis (**Figure 8E**), the intestinal injury was positively correlated with *Lachnospiraceae*_NK4A136_group, *Clostridi*a_UCG-014, and *Muribaculaceae*, but it was negatively correlated with *Escherichia-Shigella*.

## Discussion

In the medical area, *Bifidobacterium* is frequently utilized as a micro-ecological agent to control micro-ecological illnesses and preserve micro-ecological equilibrium. Meanwhile, as a physiological probiotic, it has various functions including biological barrier, anti-tumor, immune enhancement, and improvement of the gastrointestinal tract. Recent evidence showed that *Bifidobacterium* could be in intensive communication with the epithelial cells of the intestinal mucosa, forming a spatial barrier that protects the cells from colonization by pathogenic bacteria (26). Therefore, in this research by establishing an LPS-induced model of impaired intestinal mucosal immunity in mice, we investigated the enhancement of intestinal mucosal immunity by *B. longum* and its immunomodulatory mechanism.

In terms of the basic structural morphology of the intestine, we used H&E staining to clearly view the histomorphology, and it was observed that LPS extremely influenced the morphological integrity and growth of intestinal mucosa, which was consistent with the previous study (27). In the LPS group, the intestinal epithelium appeared anomalous, with an abnormal inflammatory cell infiltration and edema pattern. On the contrary, the abnormal pattern that disappeared in the *Bifidobacterium* treatment group was no significant congestion of the intestinal epithelium and occasionally congested, which was consistent with Cui et al. (28). We also quantified intestinal damage by DAI and histological scores. According to the aforementioned findings, *Bifidobacterium* may significantly lessen intestinal damage brought on by LPS, in particular in the BL-10 group. A survey by Xiang et al. discovered that *Bifidobacterium* could reduce the LPS-induced increased intestinal permeability and damage to intestinal cells (29), which was identical to our findings.

The intestinal goblet cells on the surface of the mucosa can generate mucus, which represents the main barrier limiting the contact between the host and the commensal flora and preventing microbial translocation (30). Hence, genes associated with mucus production and TJ protein expression play a critical role in gut-associated infections (31). Simultaneously, Chiu’s score and AB-PAS staining indicated alterations in the intestine (32). Elevated intestinal mucosal permeability was manifested by elevated serum D-Lac levels (33), which was also expressed in our results. The serum D-Lac content was substantially increased after 6 hours of intraperitoneal LPS injection, while it was significantly decreased in the *Bifidobacterium* treatment groups, particularly in the BL10 group. In the result of Chiu’s score, the *B. longum* BL-10 decreased with the greatest difference compared to the LPS group. Moreover, the formation of functional TJ proteins, including Claudin1, Occludin, and ZO-1, is critical for the maintenance of gut permeability and intestinal barrier function (34). In **Figure 2**, there was a clear trend of an increase in all the TJ proteins mRNA expression of the BL10 group compared with the LPS group. The research of Xiang et al. also indicated that *Bifidobacterium* pretreatment up-regulated occludin and ZO-1 expression to reduce intestinal epithelial damage, thereby enhancing intestinal barrier function. The mucus layer, which comprises mucin and trefoil factor that obstructs the pathogen colonization in the gut, is an imperative line of defense for the intestinal barrier (29, 35). The goblet-cell-specific mucin Muc 2 expression can also alter epithelial protection (36). **Figure 2** showed the Muc 2 of *Bifidobacterium* was significantly increased, which corresponded with Shashank et al. discovering higher Muc-2 mRNA expression to prevent mucosal damage (34). This study has shown that the *B.longum* BL-10 can prevent LPS damage to tight junction proteins, mucins, and goblet cells to protect the intestine’s immune function.

The immune system of the intestine consists mainly of CD4^+^ T cells, CD8^+^ T cells, DC cells, IgA plasma cells, etc., including the products they synthesize and secrete, such as cytokines (37). *Bifidobacterium* can modulate specific immune cells to enhance intestinal mucosal immunity (38). The findings indicated that LPS reduced the number of CD4^+^ T cells and CD8^+^ T cells in the intestine, with a more pronounced reduction in the number of CD4^+^ T cells. The analogical conclusions were obtained from the experiments of Zhu et al. (39). The number of CD4^+^ T cells was significantly increased in the *B.longum* BL-10 treatment group compared to the LPS group, and increased immune cells indicated enhanced immunity in mice. A previous study by Jiang et al. demonstrated that a decrease in the CD4^+^/CD8^+^ T cells ratio led to intestinal dysfunction (40). Our research indicated that *B. longum* BL-10 substantially raised the cell ratio, which demonstrated that it enhanced intestinal mucosal immunity. Additionally, in the number of IgA plasma cells study results, there was a clear trend of increase in the BL10 group compared with the BB12 group, and it is consistent with the discovery of Xie et al. (25). DCs are specialized antigen-presenting cells essential for the regulation of T cell-associated immune responses (41). As with other immune cells, the number of DC cells was significantly higher in the bifidobacteria intervention than in the LPS group. Therefore, *B.longum* BL-10 increased the number of immune cells in the intestine, including CD4+T cells, CD8+T cells, IgA plasma cells, and DC cells, to enhance intestinal mucosal immunity.

It is well known that Th1 and Th2 are the two subpopulations of activated CD4^+^ T cells’ differentiation (42). According to cytokine secretion and immune function differences, Th1 cells release the cytokines IL-2, IFN-γ, and TNF-α, which are mostly engaged in cell-mediated immunity, whereas Th2 cells secrete the cytokines IL-4, IL-6, and IL-10, which are primarily involved in humoral immunity (43). To maintain normal immune function, Th1 and Th2 should function in a dynamic balance in the body (44). Noritoshi et al. showed that *B. longum* improved the Th1/Th2 balance to enhance immunity in mice (45). The present experiment results showed that Th1-mediated cytokines were significantly adjusted compared with the LPS group after the interaction of *B. longum* BL10, especially in TNF-α and IFN-γ. In contrast, Th2-mediated cytokines including IL-4, IL-6, and IL-10 were increased, which is similar to Xie et al’s results (25). Moreover, in the reports on reducing allergic inflammation of β-lactoglobulin, *B. longum* increased Th1 cytokines and decreased Th2 cytokine production in mice. We deduced the results might be associated with the mice models and different immunoreactions. Th17 is a novel subpopulation of T-cell differentiation that has a reciprocal relationship with Th1, Th2, and Treg cells (46). Therefore, it has been suggested that there may be a Th1/Th2/Th17/Treg balance in the immune-inflammatory response (46). IL-6 and IL-17 are critical cytokines that induce differentiation of CD4^+^ T cells to Th17 cells, and IL-23 maintains stable Th17 differentiation and maturation in the late stages of differentiation (47). The results of the experiment showed an increased level of IL-17, IL-22, and IL-23 after treatment with *B.longum* BL-10 (48). These results indicated that *B.longum* BL-10 maintained the normal intestinal immune function by stimulating the secretion of cytokines and regulating the Th1/Th2 and Th17/Treg balance. T-bet, GATA-3, RORγt, and Foxp3 are key regulatory proteins for Thl, Th2, Thl7, and Treg cell differentiation respectively. The ratio of T-bet/GATA-3 and RORγt/Foxp3 is also used to reflect the Th1/Th2 and Th17/Treg balance (49). Western blot results showed that the T-bet/GATA-3 ratio of the *B. longum* BL-10 group was significantly lower than that of the LPS group. The ratio of RORγt/Foxp3 was also significantly reduced in the BL10 group compared to the LPS group. Our study clearly demonstrated that *B. longum* BL-10 regulated the body’s immune function by regulating the Th1/Th2 and Th17/Treg balance, which showed a greater impact on the Th1/Th2 balance.

An important factor in the damage caused to the intestinal mucosa by LPS is TLR4 activation (50). It has been shown that LPS damages intestinal epithelial cells directly in the intestinal mucosa and influences the expression of intestinal tight junction proteins *via* the TLR4-mediated signaling pathway (51). Thus, activating intracellular NF-κB produces large amounts of pro-inflammatory factors, triggers an inflammatory cascade *in vivo* (52), and contcontinuesexacerbate intestinal damage (53). In **Figure 7**, there was a significant trend of increase in the protein expression of TLR4 and NF-κB p65 in the LPS group, which was consistent with Li et al. (54). However, the expressions in the *B.longum* BL-10 group were significantly reduced, indicating reduced levels of inflammation in mice. This result was also verified by the conclusions of Li et al. (54). All of the analyses indicated that the *B.longum* BL-10 group was effective at inhibiting the activation of the NF-κB signaling pathway.

The dysbiosis in gut microbiota has been recently found to be associated with intestinal injury pathogenesis (55). The changes in the gut microbiota and increase of harmful metabolites (LPS) promote intestinal injury exacerbation (56). In this study, the relative abundance of *Escherichia-Shigella* was significantly increased in the LPS-induced intestinal injury mice, and pretreatment with *B. longum* BL-10 alleviated the liver injury in mice, as indicated by reducing the *Escherichia-Shigella* level. Moreover, *B. longum* BL-10 had preferable to increase *Lachnospiraceae*_NK4A136_group and *Clostridia*_UCG-014, suggesting probiotics mitigated intestinal injury by inhibiting pernicious bacteria. Therefore, the dysbiosis in gut microbiota was prospective related to the pathogenesis of LPS-induced intestinal injury, and *B. longum* BL-10 treatment relieved the gut dysbiosis and shifted it to a beneficial profile.

## Conclusion

The results of the current study showed that *B. longum* BL-10 might reduce the gut microbial imbalances and immunological damage caused by LPS in mice. Evidently, pretreatment with *B. longum* BL-10 increased the relative proportions of the Clostridia UCG-014 and Lachnospiraceae NK4A136 group while decreasing the proportions of Escherichia-Shigella. As seen by lower levels of the intestinal pro-inflammatory cytokines TNF-, IFN-γ, and IL-2 and increased production of the anti-inflammatory cytokines IL-4 and IL-10, it also modulated the TLR4/NF-B signaling pathways to alleviate the intestinal mucosal immune damage. In the end, intestinal immunity was improved by decreasing intestinal mucosal damage, increasing the number of immune cells, and modifying the immunogenic cell ratio. Therefore, the current study offered a functionally thorough evaluation of *B. longum* BL-10 as a preventative for intestinal mucosal immune damage.

## Data availability statement

The original contributions presented in the study are included in the article/**Supplementary Material**. Further inquiries can be directed to the corresponding authors.

## Ethics statement

The animal study was reviewed and approved by the Animal Ethics Committee of NEAU (ethic approval code: NEAUEC20210475).

## Author contributions

JD: Conceptualization, Methodology, Software, Writing- Reviewing and Editing. LP: Data curation, Writing- Original draft preparation. TC: Data curation, Writing- Original draft preparation. LS: Visualization, Investigation. DL: Visualization, Investigation. BL: Supervision. SW (Corresponding Author): Supervision. GH (Corresponding Author): Supervision. All authors contributed to the article and approved the submitted version.

## Funding

Present research work was financially supported by the Natural Science Foundation of Heilongjiang Province (YQ2020C013), Young Elite Scientist Sponsorship Program by CAST (YESS20200271), the National Natural Science Foundation of China (32101919), Chinese nutrition society - Feihe physique nutrition and health research fund (CNS-Feihe2020A37) and the Open Project Program of China-Canada Joint Lab of Food Nutrition and Health, Beijing Technology and Business University (BTBU), Beijing 100048, China (KFKT-ZJ-2104).

## Conflict of interest

The authors declare that the research was conducted in the absence of any commercial or financial relationships that could be construed as a potential conflict of interest.

## Publisher’s note

All claims expressed in this article are solely those of the authors and do not necessarily represent those of their affiliated organizations, or those of the publisher, the editors and the reviewers. Any product that may be evaluated in this article, or claim that may be made by its manufacturer, is not guaranteed or endorsed by the publisher.
