# Supplementary material for: Immunomodulatory effects of the Bifidobacterium longum BL-10 on lipopolysaccharide-induced intestinal mucosal immune injury
Source: Front Immunol. 2022 Aug 24;13:947755. doi: 10.3389/fimmu.2022.947755 (PMC9450040; doi:10.3389/fimmu.2022.947755)
Supplement: Supplementary file 1 [file Table_1.docx]

**Supplementary** **materials**

**Table.1** DAI score rules

| Body weight loss (%) | Stool type | Blood in feces | Score |
| --- | --- | --- | --- |
| ＜2% | Normal | Negative (no bleeding) | 0 |
| ≥2-5%＜ | Softer stool | Weak positive (weak hemoccult) | 1 |
| ≥5-10%＜ | Moderate diarrhea | Positive (visual blood in feces) | 2 |
| ≥10-15%＜ | Diarrhea | Strong positive (fresh rectal bleeding) | 3 |
| ≥15% | - |  | 4 |

**Table.2** Histological score

| Parameters | Score | Histological features |
| --- | --- | --- |
| Surface epithelial loss | 0 | No change |
| Crypt destruction | 1 | Localized and mild |
|  | 2 | Localized and moderate |
| Inflammatory cell infiltration into the mucosa | 3 | Extensive and moderate |
|  | 4 | Extensive and severe |

**Table.3** Chiu's score

| Grade | Characteristics |
| --- | --- |
| 0 | Mucosa with normal villi |
| 1 | Development of the subepithelial Gruenhagen’s space, usually at the villus apex, frequently associated with capillary congestion |
| 2 | Extension of the subepithelial space with moderate lifting of epithelial layer from the lamina propria |
| 3 | Massive epithelial lifting down the sides of the villi |
| 4 | Denuded villi with lamina propria and dilated capillaries exposed.  Increased cellularity of lamina propria may be noted |
| 5 | Digestion and disintegration of lamina propria; hemorrhage and ulceration. |

**Table.4** The sequences information of primers

| Gene | Primer sequence (5’→3’) |
| --- | --- |
| Tight junction proteins |  |
| ZO-1 | F: AACCCGAAACTGATGCTGTGGATAG |
|  | R: CGCCCTTGGAATGTATGTGGAGAG |
| Claudin1 | F: GCTGGGTTTCATCCTGGCTTCTC |
|  | R: CCTGAGCGGTCACGATGTTGTC |
| Occludin | F: TTGGCTACGGAGGTGGCTATGG |
|  | R: CCTTTGGCTGCTCTTGGGTCTG |
| MUC2 | F: CGAGCACATCACCTACCACATCATC |
|  | R: TCCAGAATCCAGCCAGCCAGTC |
| Housekeeping gene |  |
| β-actin | F: GGTTGTCTCCTGCGACTTCA |
|  | R: TGGTCCAGGGTTTCTTACTCC |
